# Se(IV)/Se(VI) adsorption mechanisms on natural and on Ca-modified zeolite for Mediterranean soils amended with the modified zeolite: prospects for agronomic applications

**DOI:** 10.1007/s11356-022-24979-2

**Published:** 2023-01-14

**Authors:** Ioannis Zafeiriou, Dionisios Gasparatos, Dafni Ioannou, Maria Katsikini, Fani Pinakidou, Eleni C. Paloura, Ioannis Massas

**Affiliations:** 1 Laboratory of Soil Science and Agricultural Chemistry, Department of Natural Resources Management & Agricultural Engineering, School of Environment & Agricultural Engineering, Agricultural University of Athens, 11855 Athens, Greece; 2 Department of Physics, Aristotle University of Thessaloniki, GR 54124 Thessaloniki, Greece

**Keywords:** Selenite, Selenate, Modified zeolite, Soil properties, Adsorption–desorption, XAFS

## Abstract

In the present study, the ability of a modified CaCl_2_ zeolite (Ca-Z) to both increase Se(IV) availability and restrict Se(VI) mobility in soils is examined. As it was resulted from batch experiments and verified by X-ray absorption fine structure (XAFS) and X-ray fluorescence (XRF) spectroscopies, higher amounts of both Se species adsorbed on Ca-Z compared to natural zeolite (Z-N) forming outer-sphere complexes while the oxidation state did not alter during agitation of samples. Thereafter, Ca-Z was incorporated in six Greek soils, divided into acid and alkaline, at a 20% (w/w) rate and a series of equilibrium batch experiments were performed with soils alone and soils-Ca-Z mixtures to investigate sorption and desorption processes and mechanisms. The acid soils, either treated with Ca-Z or not, adsorbed higher amounts of Se(IV) than alkaline ones, whereas soils alone did not adsorb Se(VI) but impressively high adsorption of Se(VI) occurred in the Ca-Z-treated soils. Desorption of Se(IV) was higher from the Ca-Z-treated soils and especially from the acid soils. Higher distribution coefficients of desorption than the distribution coefficients of sorption were observed, clearly pointing to a hysteresis mechanism. The experimental data fitted with Langmuir and Freundlich isotherms. In the presence of Ca-Z, the Langmuir *q*_*m*_ values increased indicating higher Se(IV) retention while Langmuir *b*_*L*_ values decreased suggesting lower bonding strength and higher Se(IV) mobility. Overall, treating the soils with Ca-Z increased Se(IV) adsorption and mobility whereas it provided sites for Se(VI) adsorption that did not exist in the studied soils.

## Introduction

Depending on its concentration, selenium (Se) can be a beneficial trace element or a toxic agent for plants, animals, and humans (Gupta and Gupta 2017). The range between deficiency and toxicity for the daily intake of Se in humans is very narrow as it could be defined on the basis of current estimates between 30 µg, which is considered an inadequate intake level, and 900 µg intake dose per day where it may be harmful (Winkel et al. 2015). Selenium deficiency has been linked in the literature to the onset of various health problems such as cardiovascular and myodegenerative diseases, infertility, and cognitive decline (Shreenath et al. 2021), while it is estimated that more than 1 billion people suffer from Se malnutrition. The main dietary source of Se intake is the consumption of agri-food products, in which the concentration of Se reflects the concentration and chemical form of the element in the soils (Schiavon et al. 2020).

The Se concentration in soils varies globally with the mean value estimated relatively low at 0.4 mg kg^−1^, while the usual range of concentrations is 0.01 and 2 mg kg^−1^; soils which contain a total Se concentration < 0.5 mg kg^−1^ are classified as deficient (Mirlean et al. 2017).

Selenium deficiency has been recorded in many countries around the world such as China, Canada, Spain, and USA, while Greece is also classified as deficient with reports of low Se concentration values in both arable soils and agricultural products (Sharma et al. 2016; Gupta and Gupta 2017; Zafeiriou et al. 2020). Therefore, many studies focus on the Se enrichment of agricultural soils through fertilization to achieve the production of biofortified agricultural products. The main questions in such studies have always been both the chemical form in which the element should be added and the recommended doses per crop species.

There are several reports in the literature where Se is added as a mineral fertilizer primarily as sodium selenite or sodium selenate, with the vast majority of conclusions suggesting that the most appropriate form for achieving biofortification of edible parts of plants is that of sodium selenate (Alfthan et al. 2015; Zafeiriou et al. 2022a, b). Only a few studies report greater selenite efficiency (Ros et al. 2016). This is attributed on the one hand to the faster uptake and translocation from roots to leaves and stems of the selenate and its faster transformation into organic forms, and on the other hand, to geochemical behavior of selenate (Hasanuzzaman et al. 2020). Selenate oxyanions are very mobile in the soil environment and do not strongly adsorb to soil colloids so are available for plant uptake and possible leaching. In contrast, selenite species become less mobile in the soil environment of the rhizosphere as it forms inner-sphere complexes with Fe–Mn oxides, while even in cases where it is uptaken by plants, it is mainly concentrated in the roots and not in the above-ground part (Winkel et al. 2015; Zafeiriou et al. 2022a,b). The abovementioned situation creates a very important environmental problem, as the proposed agronomic form of inorganic Se fertilizers also raises a potential increase in the degradation of natural water resources and especially aquifers. Therefore, many researchers are opening the debate on possible solutions using soil amendments to reduce the potential leaching of Se oxyanions and other nutrients present in the soil environment in the form of mobile anions. As reported in the studies conducted by Peak (2006), Rovira et al. (2008), Mitchell et al. (2013), and Goldberg (2014a, b), iron oxides mainly and aluminum oxides as well as clay minerals were tested for their ability to retain Se oxyanions. The recent review by Zoroufchi Benis et al. (2022) summarizes the efficacy of 112 materials to adsorb Se.

Natural zeolites are widely applied to manage cationic element concentration in soils but have been rarely used for anion adsorption because they have a negatively charged surface caused by isomorphic substitution in the crystal lattice (Nakhli et al. 2017). For anion management, as selenite and selenate, modification of the zeolite surface is necessary to make the adsorbent more efficient for sorption (Bilici Baskan and Pala 2011; Suazo-Hernández et al. 2019). Ca-modified zeolite was used by many researchers for anion removal from aqueous solutions (Zhang et al 2011; Hermassi et al 2016; You et al 2017; Mitrogiannis et al 2017). To our knowledge, however, Ca-modified zeolites have not been tested for their effect on added Se behavior in soils regarding adsorption and desorption rates of Se(IV) and Se(VI).

This study was initiated by a consideration relative to the optimal utilization of an effective amendment that could be able to increase Se retention when added in soils and to gradually release it in the soil solution (especially that of Se (VI)), thus restricting toxicity and leaching problems on one hand and controlling the availability of the element for plant uptake on the other.

The preliminary approach was accomplished through a series of equilibrium batch experiments that were performed to address the following issues: (1) adsorption of Se(IV) and Se(VI) on a natural zeolite (Z-N) and on its modified by CaCl_2_ form (Ca-Z). For the investigation of adsorption mechanisms, X-ray absorption fine structure/X-ray fluorescence (XAFS/XRF) spectroscopy techniques were used. XAFS spectroscopy is particularly suited for the study of the sorption mechanisms of metal or non-metal oxyanions in soils as well as for the determination of alterations in their oxidation state (Favorito et al. 2017; Mo et al. 2021). (2) The adsorption characteristics of Se(IV) and Se(VI) on typical Greek soils with contrasting properties, (3) the incorporation of a CaCl_2_-modified natural zeolite into the studied soils in order to evaluate its ability to control adsorption/desorption processes of Se(IV) and Se(VI), and (4) the reversibility of the adsorption process both in soils alone and in the soil-Ca-Z mixtures by utilizing the desorption data.

## Materials and methods

### Chemicals

All the reagents used in this study were standard analytical grade or better. Stock solutions of Se(IV) and Se(VI) were prepared from sodium selenite (NaSeO_3_) and sodium selenate (NaSeO_4_), respectively, purchased from Arcos Organics. Deionized water (Milli-Q; Millipore) was used in every solution. All the glassware used in the experiments was first soaked for 24 h in 10% (v/v) nitric acid and finally rinsed three times with deionized water.

### Soils

The soil samples used in this study were collected from the surface layer (0–10 cm depth) of alluvial soils from six different sites with agricultural land use of Peloponnese Region (Greece). The soil samples were air dried at room temperature, ground, and passed through a 2-mm sieve. Then, they were stored in self-sealed polyethylene bags for further analysis. These samples were selected on the basis of their acidity values as pH significantly affects sorption phenomena in the soil system. Selected properties of soil samples are presented in Table 1. Soil pH and EC (electrical conductivity) were measured in a soil/water ratio 1:1 (w/v), using automated pH (Selecta-2005) and EC (Selecta-2005) meter, respectively (Page et al. 1982). Particle size distribution was measured by using the hydrometer method (Bouyoucos 1951). Cation exchange capacity (CEC) was quantified by Na-acetate method at pH 7.0 (Page et al. 1982), and total organic carbon (TOC) was obtained by the Walkley–Black wet oxidation method (Nelson and Sommers 1982). Calcium carbonate equivalent (CCE) was estimated by using a digital calcimeter (FOG L; bd INVENTIONS, Greece) (Bilias and Barbayiannis 2017), while active carbonate fraction was measured equilibrating the soil sample with ammonium oxalate following the method described by Loeppert and Suarez (1996). Free and amorphous Fe, Mn, and Al oxide contents were determined after extraction by dithionate-citrate-bicarbonate (DCB) method (Gasparatos et al. 2004), and by acid ammonium oxalate method in dark conditions (Gasparatos et al. 2006), respectively. Fe, Al, and Mn concentrations in the extractants were quantified by atomic absorption spectrophotometry, AAS spectrometry Varian AA240FS. Total Se concentration was extracted by aqua regia (HCl/HNO_3_, 3:1) (Gasparatos and Haidouti 2001). The extractants were analyzed for dissolved Se concentrations by inductively coupled plasma–mass spectroscopy (ICP-MS, ThermoiCAPQc; Thermo Fisher Scientific).

**Table 1:** Physicochemical properties of the studied soils

Soil	1	2	3	4	5	6
Soil properties						
Clay (%)	24	17	22	26	22	26
Silt (%)	30	21	22	52	40	34
Sand (%)	46	62	56	22	38	40
pH (1:1)	6.23	5.46	5.74	7.35	7.5	7.4
CaCO_3_ eq. (%)				9.8	9.3	8.4
Act. CaCO_3_ (%)				1.2	1.4	1.4
EC (μS cm^−1^)	258	626	172	277	357	380
O.C. (%)	1.08	0.66	0.9	2.16	0.96	1.32
Fe_d_ (%)	2.85	1.81	2.76	1.46	1.45	1.06
Fe_o_ (%)	0.24	0.18	0.18	0.07	0.04	0.12
Al_d_ (%)	0.23	0.09	0.20	0.09	0.01	0.10
Al_o_ (%)	0.13	0.08	0.13	0.12	0.01	0.12
Mn_d_ (%)	0.81	0.26	1.06	0.21	0.18	0.39
Mn_o_ (%)	0.62	0.20	0.82	0.14	0.15	0.36
Se total (mg kg^−1^)	0.19	0.14	0.17	0.30	0.22	0.24

*EC* electrical conductivity; *OC* organic carbon; *Act. CaCO*_*3*_ active carbonates; *Fe*_*d*_, *Al*_*d*_, *Mn*_*d*_ free oxides; *Fe*_*o*_, *Al*_*o*_, *Mn*_*o*_ amorphous oxides

### Experimental procedure

#### Zeolite material

Natural zeolite (Z-N) used in this experiment was derived from S&B Industrial Minerals S.A., in Greece, with a particle size < 0.15 mm. X-Ray diffraction analysis had showed that it was mainly constituted of clinoptilolite (> 85%) with small impurities of cristobalite, feldspar, dolomite, micas, and clays. The cation exchange capacity (CEC) was 180 cmol_c_ kg^−1^. According to XRF analysis, the chemical composition of the Z-N was SiO_2_ 70.08%, Al_2_O_3_ 11.72%, TiO_2_ 0.14%, Fe_2_O_3_ 0.67%, MgO 0.71%, CaO 3.18%, Na_2_O 0.55%, K_2_O 3.50%, and LOI 9.45% (Anastopoulos et al. 2012).

#### Z-N modification

Since impurities in the pores of natural zeolites may block its sorption ability (Kragović et al. 2012), Z-N used in this study was chemically modified before being applied to the experiments. In this stage, N-Z was washed by DI water three times, dried at 60 °C for 24 h. Then, 20 g of washed Z-N was dispersed in 300 mL of 0.5 M CaCl_2_ solution by magnetic stirring for 24 h, at room temperature. After stirring, the mixture was centrifuged and the residue washed appropriate times with DI water until excess salt entrapped within zeolite sample was removed, as it was tested by AgNO_3_ titration for existence of chloride ions in the rinse. Afterwards, the material was dried at 60 °C and stored in a clean glass flask for use in the adsorption/desorption experiments. The produced modified Z-N named as Ca–zeolite and symbolized as Ca-Z.

#### Selenium adsorption on Z-N and Ca-Z

To test if the modification of natural zeolite resulted to increased sorption capacity, 1 g of Z-N and Ca-Z were equilibrated for 24 h with 30 mL of solution containing 40 mg Se L^−1^ in the form of either Na_2_SeO_4_ or Na_2_SeO_3_ (the procedure followed is the same as the one described in “Soil and soil-Ca-Z adsorption/desorption experiments” section). The results showed higher adsorption of both Se species on the modified zeolite and thus it was decided to test Ca-Z in a mixture with different soils in order to thoroughly examine the effect that the Ca-Z might have on the soils’ ability to adsorb and to desorb Se, and thus to regulate to an extent the concentration of added Se in the soil solution and the available Se for plant uptake. However, to answer questions relative to Se speciation and the type of bonding on the solid material (i.e., outer-sphere, inner-sphere complexes) that are raised during equilibrium batch experiments, Z-N and Ca-Z samples equilibrated with Se(IV) and Se(VI) were analyzed with XAFS spectroscopy.

#### XAFS/XRF spectroscopy

The oxidation state and the coordination environment of Se after its sorption by the natural and modified zeolite were studied by XAFS spectroscopy. The measurements were conducted at the KMC-II beamline of the BESSY-II storage ring of the Helmholtz Zentrum Berlin (Többens and Zander 2016). The spectra were recorded in the fluorescence yield mode using a XFlash-Bruker energy-dispersive detector that allows the energy discrimination of the Se K_α_ fluorescence signal. The spectra were normalized using the signal of an ionization chamber positioned in front of the sample. The angle of incidence was 45° and the fluorescence detector was positioned on the horizontal plane at right angle to the beam. Na_2_SeO_4_ and Na_2_SeO_3_ powder samples were used for reference purposes. The X-ray fluorescence (XRF) spectra were recorded using the same experimental set-up. An excitation energy of 13 keV was used.

#### Soil and soil-Ca-Z adsorption/desorption experiments

Batch agitation experiments were performed to evaluate Se retention from soil samples as a function of initial selenite and selenate concentrations. All experiments were carried out at room temperature (20 ± 1 °C), in 50-mL sealed falcon tubes, under mechanical shaking in a reciprocal shaker. Adsorption experiments were performed in two sets. In one set, only soil was used as an adsorbent, while in the other set, a soil mixture with 20% Ca-Z (w/w) was used. Sorption isotherms were produced by agitating 1 g of adsorbent with 30 mL of Se solution at different Se rates (1, 5, 10, 20, 30, 40, and 60 mg L^−1^), in the form of NaSeO_3_ and NaSeO_4_, respectively. Adsorbent falcon tubes were shaken for 24 h. At that time interval, adsorption equilibrium was achieved as tested by previous experiments (Zafeiriou et al. 2020). Then, the samples were centrifuged at 3000 × *g* for 15 min, the supernatant was filtered through Whatman filter paper No. 42, and the filtrate was analyzed for Se using ICP-MS (ThermoiCAPQc; Thermo Fisher Scientific).

The amount of Se adsorption at equilibrium, was calculated from the difference between the initial and final concentration of Se, as described in Eq. (1):

1 $${q}_{e}=\frac{\left({C}_{i}-{C}_{e}\right)\times V}{m}$$

where ${q}_{e}$ is the adsorption capacity (mg g^−1^), ${C}_{i}$ is the initial Se concentration (mg L^−1^), ${C}_{e}$ is the equilibrium Se concentration (mg L^−1^), *V* is the volume of solution (L), and $m$ is the mass of the adsorbent (g). The percentage of adsorption or sorption efficiency (SE) can be calculated using Eq. (2):

2 $$Ads\left(\%\right)=SE =\frac{{C}_{i}-{C}_{e}}{{C}_{i}}\times 100$$

Desorption experiments were performed immediately after sorption experiments for all concentrations, species, and adsorbents, using 0.1 M KCl as desorbing agent (Dhillon and Dhillon 1999; Zafeiriou et al. 2020). To desorb sorbed Se, all samples retained after Se sorption and agitated with 30 mL of 0.1 M KCl solution, following the same procedure of shaking, centrifugation, and filtration, as in the adsorption experiments. The concentration of Se desorbed was checked in the filtrate by ICP-MS. The percentage of Se desorbed was calculated by the following Eq. (3):

3 $$Desorption\left(\%\right)=\frac{{q}^{^{\prime}}}{{q}_{e}}\times 100$$

where ${q}_{e}$ and ${q}^{^{\prime}}$ is the adsorption and desorption capacity (mg Se g^−1^) at equilibrium, respectively. ${q}^{^{\prime}}$ is calculated as ${C}_{des}\times (\frac{V}{m})$, where ${C}_{des}$ (mg L^−1^) is the Se concentration in the desorbing solution at equilibrium.

All batch adsorption and desorption experiments were performed in duplicate and the mean values were taken for further calculation.

#### Equilibrium data

##### Adsorption distribution coefficient $({K}_{d})$–desorption distribution coefficient $({K}_{{d}^{^{\prime}}})$

The distribution coefficients values (${K}_{d}\mathrm{ and }{K}_{{d}^{^{\prime}}}$) were determined by using the following formulas:

4 $${K}_{d}={~}^{{q}_{e}}\!\left/ \!{~}_{{C}_{e}}\right.$$

5 $${K}_{{ }^{^{\prime}}{d}^{^{\prime}}}={~}^{{q}_{des}}\!\left/ \!{~}_{{C}_{des}}\right.$$

where ${q}_{e}$ (mg kg^−1^) is the amount of Se adsorbed per kilogram of adsorbent, ${C}_{e}$ (mg L^−1^) is the equilibrium concentration of Se in the solution, and ${q}_{des}$ and ${C}_{des}$ are the respective equilibrium values derived from desorption data ${q}_{des}={q}_{e}- {q}^{^{\prime}}$

Hysteresis coefficient (HC) was calculated according to Eq. (6) (Shirvani et al. 2006—Chemosphere):

6 $$HC=\left({~}^{{K}_{{d}^{^{\prime}}}-{K}_{d}}\!\left/ \!{~}_{{K}_{{\mathrm{d}}^{^{\prime}}}}\right.\right)\times 100$$

##### Isotherms

Isotherm models are widely applied in equilibrium adsorption data to provide information about sorption capacity of the adsorbents under experimental conditions and to understand sorption mechanisms (Giannakopoulou et al. 2012). The Se adsorption equilibria were evaluated according to the following two-parameter isotherm models: Langmuir and Freundlich.

Langmuir adsorption isotherm, which assumes monolayer coverage of the surface and negligible interaction forces between adsorbed atoms, is expressed as

7 $$\frac{{C}_{e}}{{q}_{e}}=\frac{1}{{b}_{L}{q}_{m}}+\frac{{C}_{e}}{{q}_{m}}$$

where ${q}_{m}$ (mg g^−1^) is the maximum adsorption capacity of the adsorbent for Se, assuming monolayer coverage sorption onto the adsorbent containing a finite number of uniform adsorption sites, and ${b}_{L}$ (L g^−1^) is the Langmuir equilibrium or affinity constant related to the energy of adsorption (affinity of binding sites). ${q}_{m}$ and ${b}_{L}$ were calculated from the slope and intercept of the linear graph of $\frac{{C}_{e}}{{q}_{e}}$ versus ${C}_{e}$. In addition, the dimensionless constant ${R}_{L}$ (Eq. 8), known as separation factor or equilibrium parameter (Vargas 2011; Constantino 2018), was determined according to the following equation (Nnaji et al. 2021):

8 $${R}_{L}=\frac{1}{1+{b}_{L}x{C}_{i}}$$

where ${C}_{i}$ is the initial Se concentration (mg L^−1^).

${R}_{L}$ is used to verify the feasibility of sorption process in the studied system, indicating that if ${R}_{L}$>1,${R}_{L}$=1, ${R}_{L}$<1, or ${R}_{L}$=0, sorption is unfavorable, linear, favorable, or irreversible, respectively.

The Gibbs free energy, Δ*G*^o^, was calculated as follows:

9 $${\mathrm{\Delta G}}^{\mathrm{o}}=-\mathrm{RTln}({b}_{L})$$

where *R* is the universal gas constant (J K^−1^ mol^−1^), *T* is the temperature (K), and *b*_*L*_ (L g^−1^) is the affinity constant of Langmuir isotherm. Negative Δ*G*^o^ values indicate that the sorption process is spontaneous (Nakamaru and Sekine 2008; Sahmoune 2018).

Freundlich adsorption isotherm is used to describe adsorption on heterogeneous surfaces and assumes that multilayer adsorption is possible (Foo and Hameed 2010)). The linearized form is expressed as

10 $${\mathrm{log}q}_{e}={\mathrm{log}K}_{F}+\left(\frac{1}{n}\right){\mathrm{log}C}_{e}$$

where *K*_*F*_ (mg g^−1^) is the Freundlich equilibrium constant related to the temperature and is regarded as quantity parameter, i.e., quantity of Se retained on the soil and soil–zeolite surfaces when selenite or selanate concentration is 1 μΜ, and *n* is a characteristic constant for the adsorbent-adsorbate system indicating the intensity of the adsorption. According to Vargas et al. (2011), *n* is the heterogeneity factor and may be used to show whether the adsorption is linear (*n* = 1), whether it is a chemical process (*n* < 1), or whether a physical process is favorable (*n* > 1). On the contrary, values of 1/*n* < 1 and 1/*n* > 1 suggest a normal Langmuir isotherm and cooperative adsorption, respectively.${K}_{F}$ and *n* were calculated from the slope and intercept of the linear graph of ${\mathrm{log}q}_{e}$ versus ${\mathrm{log}C}_{e}$. In addition, when 0 < 1/*n* < 1, the adsorption is favorable at studied conditions (Foo and Hameed 2010).

### Statistics

For statistical analysis, STATISTICA software version 10 was used.

## Results and discussion

### Soil characteristics

Considering that soil properties as pH, clay, and secondary iron oxide content greatly influence Se behavior in soil ecosystems, the soils were selected to reflect distinct differences in those properties (Table 1). Following the pH criterion, soils 1, 2, and 3 are acid while soils 4, 5, and 6 showed slightly alkaline pH values. Amorphous and free iron oxide content were considerably higher in acid soils compared to alkaline whereas no clear differences were observed for clay fraction. Other soil characteristics that may affect Se speciation, mobility, and distribution in various chemically active soil fractions are organic matter and redox potential and to a lesser extent carbonate and Al and Mn oxide content (Zafeiriou et al. 2022b). Organic matter content was low in all soils and this combined with the effective soil aeration throughout the year points to that oxidizing conditions prevail under Mediterranean conditions (Gasparatos et al. 2011). No clear differences of Al oxide amount between the six soils were detected while a trend for increased Mn oxide concentrations in acid soils was observed.

### XAFS/XRF analysis of Z-N and Ca-Z samples equilibrated with Se(IV) and Se(IV)

#### XAFS spectroscopy

**Fig. 1:**
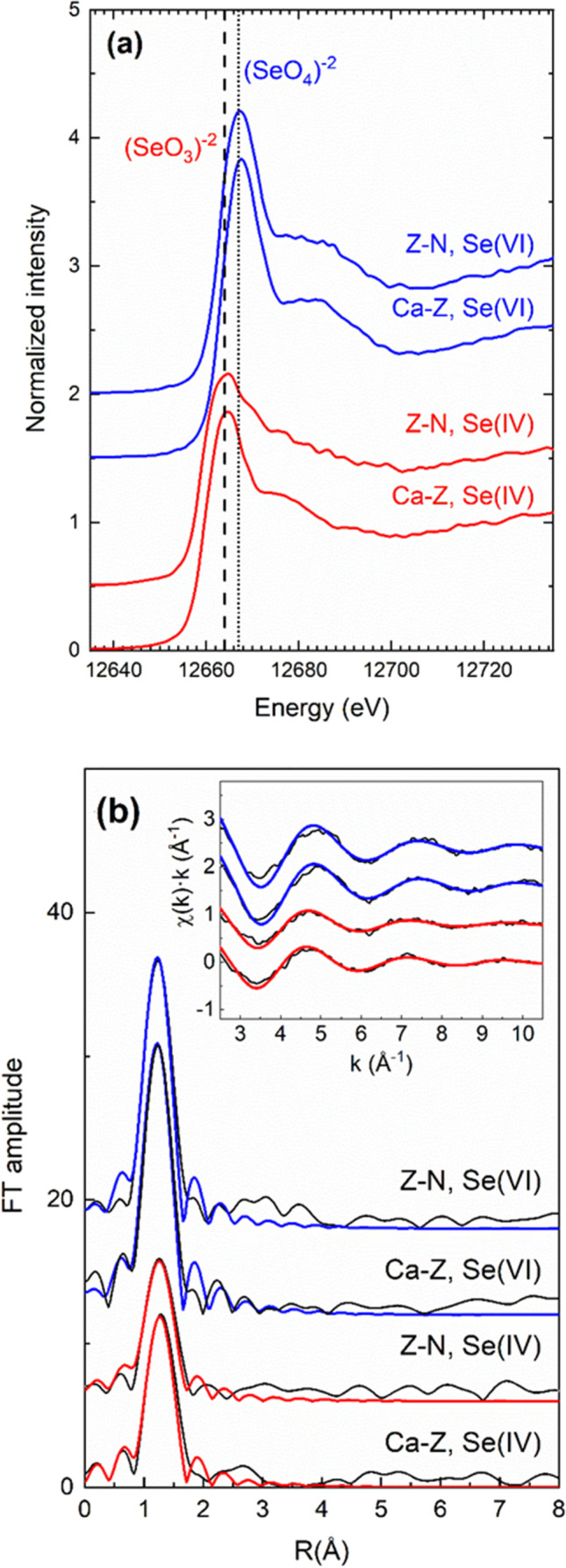
Se-K-edge XAFS spectra of the natural (Z-N) and CaCl_2_-treated (Ca-Z) zeolite samples after the adsorption of selenite, Se(IV), and selenate, Se(VI). **a** XANES spectra normalized to the edge jump. Dashed and dotted vertical lines denote the position of the white lines for Se(IV) and Se(VI), respectively. **b** Fitting of the EXAFS spectra in R-space (Fourier transform, FT) and k-space, χ(k), that is shown in the inset. Thin black and thick colored lines correspond to the experimental and fitting curves, respectively

The Se-K-edge X-ray absorption near edge structure (XANES) and extended XAFS (EXAFS) spectra of the natural and CaCl_2_ modified zeolites subjected to adsorption of selenate or selenite are shown in Fig. 1a and b, respectively. The most prominent feature of the XANES spectra is the intense peak (white line) at ca. 12,665 eV which is assigned to 1s→4p transitions of the Se atom. The white line of the Se(IV) and Se(VI) species exhibits an energy separation of 3 eV and it is a useful probe for the determination of the oxidation state of Se (Nothsteinet al. 2016; Qin et al. 2017; Christensen et al. 2004). It is evident, from Fig. 1a, that the oxidation state of Se does not change after its adsorption on the natural or CaCl_2_ treated zeolite. The oxidation of Se(IV) to Se(VI) during sorption on K_2_FeO_4_ in aqueous solution has been reported (Xu and Fu 2020).

The Se-K-edge EXAFS spectra were subjected to subtraction of the atomic background, normalization to the edge jump, and transformation from the energy to the k-space using the ATHENA software (Ravel and Newville 2005). The resulted χ(k) spectra were fitted using the photoelectron scattering paths constructed with the FEFF8 code (Ankudinov et al. 1998). Representative fitting of the EXAFS spectra in the k- and R-space is shown in Fig. 1b. Only one path (Se-O) was necessary for the fitting, revealing that in all cases, outer-sphere complexes are formed. The nearest-neighbor distances of Se with O in the Se oxyanion were found equal to 1.69–1.70 ± 0.01 Å and 1.64–1.65 ± 0.01 Å for the zeolite (natural and modified) which adsorbed Se(IV) and Se(VI), respectively. The corresponding coordination numbers were found equal to 2.7–2.8 ± 0.5 and 4.0–4.1 ± 0.5, respectively. Both the Se-O distances and the coordination numbers verify that alteration in the oxidation state of Se upon adsorption does not take place (Nie et al. 2017; Peak et al. 2006).

#### X-Ray fluorescence analysis

**Fig. 2:**
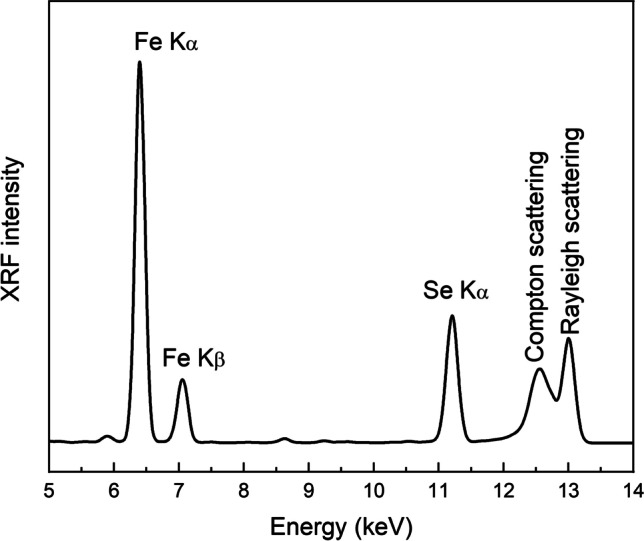
Representative XRF spectrum of the sample Ca-Z, Se(IV) recorded using an excitation beam with energy 13 keV

X-Ray fluorescence (XRF) spectra were recorded with excitation energy of 13 keV. The areas under the peaks related to emission of Fe and Se were used for the determination of the Se/Fe concentration ratio in the zeolite samples. A representative spectrum is shown in Fig. 2. Since the powder samples were fixed using Kapton tape that causes reduction of the fluorescence signal for low energy emission peaks, the relative concentration of Se was determined using the Fe peak that lies close to the fluorescence peak of Se. The sensitivity factors of Fe and Se were determined using the fluorescence yield, absorption cross section, jump ratios, and emission probabilities obtained from the programs Mucal on the web (P. Bandyopadhyay and C.U. Segre, http://www.csrri.iit.edu/mucal.html) and HEPHAESTUS. The results are listed in Table 2. Assuming that the CaCl_2_ treatment of the zeolite does not affect its Fe content, the treatment results in an increase of the amount of Se adsorbed on the zeolite by 67% and 39% in the case of Se(IV) and Se(VI), respectively.

**Table 2:** Se/Fe ratio in g/g and wt.% of Se in the zeolite assuming that the Fe_2_O_3_ content of the zeolites is 0.67% (wt./wt.) (see the “Soils” section)

Sample	Se/Fe (g g^−1^)	wt.% of Se in the zeolite
Z-N, Se(IV)	0.064 ± 0.001	0.030 ± 0.001
Ca-Z, Se(IV)	0.108 ± 0.002	0.050 ± 0.002
Z-N, Se(VI)	0.050 ± 0.001	0.023 ± 0.001
Ca-Z, Se(VI)	0.069 ± 0.001	0.032 ± 0.001

The quoted errors originate from the fitting uncertainties in the determination of the area under the considered peaks

### Selenium adsorption onto soils and soils-Ca-Z

**Fig. 3:**
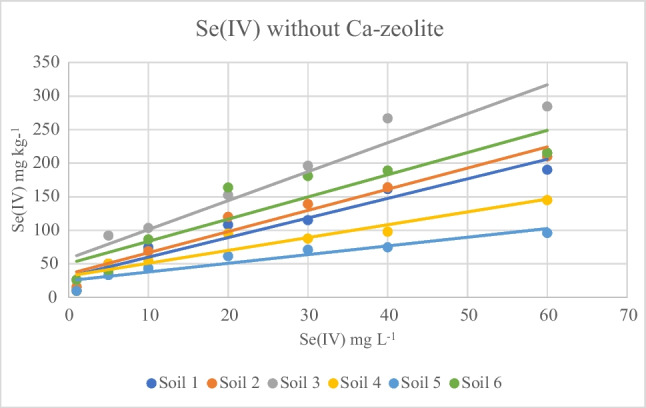
Se(IV) adsorption onto six soils, at various initial Se(IV) concentrations. Contact time 24 h, agitation rate 125 rpm, sorbent/solution ratio 1 g/0.03 L, Se concentrations at start time from 1 to 60 mg/L, temperature 22 °C. Equilibrium solution pH values 6.0–6.5

The Se(IV) adsorption onto soils in the absence of Ca–zeolite at various initial Se(IV) concentrations is shown in Fig. 3. The sorbed amount of Se(IV) increased as the initial Se(IV) concentration in the solution increased. More specifically, selenite adsorption ranged between 9.63 and 190.2 mg kg^−1^ for alkaline soils, while in the case of acid soils the corresponding values ranged between 15.75 and 284.4 mg kg^−1^ indicating an increasing trend of adsorption compared to alkaline soils.

Se(VI) adsorption on the studied soil samples was not found or it was negligible since selenate forms weak outer-sphere complexes with soil colloids resulting in increased mobility of this species. Such observation is commonly referred by many researchers like Neal and Sposito (1989) who did not detect Se(VI) adsorption over the pH range of 5.5–9.0 on four alluvial soils. However, Loffredo et al. (2011) report Se(VI) adsorption on two soils either by the formation of outer-sphere complexes on goethite or by inner-sphere complexes followed by reduction mechanisms, probably initiated by microorganisms.

The presence of Ca–zeolite affected the adsorption of Se(IV) in both acid and alkaline soils. The adsorption for alkaline soils ranged between 12.33 and 190 mg kg^−1^, giving a lower maximum value, while for acid soils the adsorption values ranged between 15.3 and 307.8 mg kg^−1^, giving in most cases marginally higher values compared to the values recorded in the absence of Ca–zeolite.

The effect of Ca–zeolite on Se(VI) adsorption was impressive as it led to a significant increase in adsorption values for both acid and alkaline soils. With respect to alkaline soils, the adsorption values ranged between 15.6 and 109.2 mg kg^−1^, while for acid soils they ranged between 14.1 and 113.1 mg kg^−1^.

Depending on added Se(IV) concentrations in the absence of Ca–zeolite, distribution coefficient (*K*_*d*_) values were within the ranges of 4–211.9 and 1.7–16.2 L kg^−1^ for acid and alkaline soils, respectively (Table 3). Over the whole range of added Se(IV) concentrations, the *K*_*d*_ values of alkaline soils were considerably lower than those of the acid soils. Liang and Li (2016) for 18 soils from China report a strong negative correlation between *K*_*d*_ and soil pH values indicating stronger adsorption of Se(IV) in acid soils. A similar pattern was formed in the presence of Ca–zeolite for Se(IV) adsorption with *K*_*d*_ values ranging between 4.2 and 189 for acid soils and 3.1 and 47.1 for alkaline soils. Regarding Se(VI) adsorption in the presence of Ca–zeolite, *K*_*d*_ values ranged between 1.7 and 58.2 for acid soils and 1.3 and 77.1 for alkaline soils.

**Table 3:** *K*_*d*_ and *K*_*d*΄_ values

Soil	*K*_*d*_			*K*_*d*′_		
	Se(IV)	Se(IV)-Z	Se(VI)-Z	Se(IV)	Se(IV)-Z	Se(VI)-Z
1	211.9–4.1	170.0–6.0	58.2–1.7	356.5–81.4	235.6–170.7	588.8–57.1
2	33.2–4.0	31.2–4.2	39.8–2.0	188.8–147.6	420.0–90.2	241.4–75.9
3	204.4–5.6	189.0–6.2	26.6–1.8	1714.0–198.6	1408.0–97.1	492.2–50.1
4	14.2–2.6	21.7–3.1	32.5–1.3	93.5–35.1	217.136.2	442.7–27.8
5	14.4–1.7	47.1–3.4	63.8–1.6	60.0–14.3	224.6–45.7	673.5–50.0
6	16.2–3.5	20.9–4.7	77.1–1.9	124.4–58.1	73.8–55.1	460.9–75.8

Ranges correspond to the values calculated for the lowest and the highest initial Se solution concentrations. *Z* stands for the modified zeolite treatments

As the concentration of the element in the solution increases, a trend of decreasing values of *K*_*d*_ is often observed, indicating that proportionally lower percentage of the added element has the potential to adsorb on the soil colloids. Indeed, for all studied soils and regardless of the Se species and the presence or not of Ca–zeolite, *K*_*d*_ values had a decreasing trend with increasing concentration of the element in the solution.

As it was shown by the XAFS spectra, the Se speciation did not alter during the adsorption batch experiments, a result that it is also supported by the pH values of the equilibrium solutions that did not change considerably compared to the initial solution pH values.

### Selenium desorption from soils and soils–Ca-Z mixture

Opposing to sorption studies, relatively little information on desorption of anions from soils or soil constituents is available in the literature. Desorption controls the release rate of the adsorbed element and hence regulates its mobility in the soil. Soil may be regarded as a medium that initially adsorbs Se but may then release it back to the soil solution. In the present study, 0.1 M KCl was used to extract both Se(IV) and Se(VI) species from the adsorbent. As it is stated by Wang et al. (2012) and Dhillon and Dhillon (1999), non-specifically Se species can be desorbed by chloride ions through mass action mechanisms and ion exchange. In the absence of Ca–zeolite, the desorption for alkaline soils ranged between 2.04 and 66.78 mg kg^−1^ while the corresponding range recorded for acid soils was between 0.45 and 58.02 mg kg^−1^. In most cases, desorption was higher for alkaline soils giving higher equilibrium desorption values when 60 mg L^−1^ was applied. The presence of Ca–zeolite affected the Se(IV) amount desorbed from the soils, as for both alkaline and acid soils the amount of selenium desorbed was higher than in the absence of Ca–zeolite, with values recorded ranging between 1.53 and 86.64 mg kg^−1^ and 0.54 and 72.66 mg kg^−1^ for alkaline and acid soils, respectively. Regarding Se(VI), a desorption pattern was obtained only in the presence of Ca–zeolite, as no adsorption occurred in its absence. The values recorded ranged between 0.87 and 40.05 mg kg^−1^ for alkaline soils while the corresponding variation for acid soils was between 0.81 and 38.19 mg kg^−1^.

The percentage of the amount of Se(IV) desorbed in the presence of the modified zeolite was higher in most cases compared to the corresponding percentage in the absence of the mineral as shown in Fig. 6. This trend is mainly evident in the cases of acid soils, a phenomenon explained by the fact that part of the total amount of oxides was replaced by Ca–zeolite (i.e., as a consequence of lower soil participation in the absorbent since 0.2 g soil was replaced by Ca–zeolite). Thus, while in one case Se(IV) ions formed mostly inner-sphere complexes with metal oxides (Natasha et al. 2018), in the other case they also formed outer sphere complexes with the modified mineral; hence, the excess desorption is due to the higher mobility of selenium in the presence of zeolite. This effect was not so apparent for alkaline soils, as in these cases the higher pH already weakened the intensity with which the oxides had adsorbed selenite ions (Balistrieri et al. 1987; Lopes et al. 2017), leading to easier desorption and similar percentage desorption values. The percentage desorption of Se(VI) followed a similar pattern to that of Se(IV). However, it is worth to note that at low rates of added Se(VI), a very small amount of the adsorbed amount was released. In fact, an approximate 4–6% and 8% desorption was observed for 1 and 5 mg Se(VI) L^−1^ rates, respectively.

Desorption distribution coefficient (*K*_*d’*_) was higher than the sorption coefficient (*K*_*d*_) for both Se species and for both soils and Ca-Z amended soils and all initial solution concentrations and decreased as the solution concentration increased (Table 3). According to Shirvani et al. (2006), since *K*_*d*_ is the ratio of adsorbed Se to Se in the equilibrium solution and *K*_*d’*_ is the ratio of adsorbed Se remained on the solid to Se in the desorption equilibrium solution, the higher *K*_*d’*_ values indicate a hysteresis mechanism. These authors attributed to hysteresis the observed higher desorption *K*_*d*_ values compared to the corresponding values for sorption, providing as explanation that the affinity of sorbate to the solid phase increased when the equilibrium reached from desorption direction.

The hysteresis coefficient (HC) is an indicator of the adsorbent capability to retain the adsorbed ions when desorbing forces are applied and thus higher HC values suggest higher hysteresis. According to Galunin et al. (2014), the ability of a soil or sediment that has been driven to its maximum sorption capacity to continue to hold the sorbed metal, even though the *C*_*eq*_ tends to zero and desorption is practically absent, is reflected with a positive hysteresis index. For all soils, hysteresis coefficient (HC) for Se(IV) and Se(VI) increases from the lower to higher initial solution concentration regardless of the presence or absence of Ca–zeolite in the solid phase (Table 4).

**Table 4:** *R*_*L*_, H.C., and Δ*G*^o^ values

Soil	*R*_*L*_			H.C			Δ*G*^o^ (kJ/mol)		
	Se(IV)	Se(IV)-Z	Se(VI)-Z	Se(IV)	Se(IV)-Z	Se(VI)-Z	Se(IV)	Se(IV)-Z	Se(VI)-Z
1	0.91–0.15	0.98–0.08	0.89–0.12	40.54–94.98	27.85–96.48	90.11–97.09	− 21.73	− 23.63	− 22.38
2	0.94–0.20	0.97–0.24	0.92–0.16	82.43–97.70	92.57–95.36	83.53–97.35	− 20.92	− 20.62	− 21.63
3	0.90–0.13	0.99–0.16	0.89–0.12	88.08–97.17	86.58–93.63	94.60–96.40	− 22.23	− 22.01	− 22.45
4	0.93–0.17	0.96–0.14	0.88–0.11	84.83–92.52	89.99–91.56	92.66–95.16	− 21.34	− 20.46	− 22.64
5	0.91–0.15	0.94–0.16	0.93–0.17	76.04–88.16	79.02–92.11	90.53–96.87	− 21.75	− 21.62	− 21.35
6	0.95–0.24	0.97–0.28	0.92–0.16	87.02–93.90	71.23–91.39	83.26–97.44	− 20.37	− 20.09	− 21.59

The two values that indicate the *R*_*L*_ and H.C. ranges correspond to the values calculated for the lowest and the highest initial Se solution concentrations. *Z* stands for the modified zeolite treatments

*H.C.* hysteresis coefficient

This might be expected since the same concentration and quantity of desorbing solution was used resulting to lower desorbing efficiency as the amount of sorbed Se increased. Reaching the maximum retaining capacity of the tested soils (i.e., equilibrium), Se at low concentrations was preferably adsorbed on high affinity sites and the surface coverage of the solid phase is low. On the contrary, at high concentrations, beyond the high affinity sites, Se was also adsorbed on lower affinity sites and the surface coverage at equilibrium was much higher. Hence, considering absolute values, the amount of Se adsorbed at low initial solution concentrations is small compared to the higher initial solution concentrations leading to higher desorption hysteresis for the higher concentrations.

From an agronomic point of view, the desorbing patterns of Se species adsorbed on soils with contrasting characteristics and on the Ca–zeolite amended soils provide strong indications that the modified zeolite can be applied into the soils to control Se availability for plant uptake. At low concentrations that are important in terms of biofortification, Ca–zeolite may assist the mobility of Se(IV) and beyond its ability to offer adsorption sites for Se(VI), otherwise absent from the soils, can regulate the release of the retained Se(VI). The latter can be regarded as an important finding because although Se(VI) is the preferable species for uptake by plants, it is not recommended for application in Se-deficient soils since it is not retained by soil colloids and leaching is inevitable driving to serious environmental implications. Furthermore, the addition of Ca–zeolite in soils with high Se(VI) concentrations may reduce toxicity effects in the soil ecosystem and in plants.

## Isotherm’s interpretation

The experimental data obtained regarding the adsorption of Se(IV) on soils fitted well with Freundlich and Langmuir isotherms, in agreement with the results of Zafeiriou et al. (2020) and Dhillon and Dhillon (1999), regardless of the presence or not of Ca–zeolite (Table 5). The calculated adsorption maxima (*q*_*m*_) from the Langmuir isotherm were in most cases higher for acid soils. A similar trend was also observed for the values of the bonding constant (*b*_*L*_), indicating that lower pH values in the soil solution led to stronger binding of Se (IV), a conclusion in agreement with many studies. The *q*_*m*_ values showed a significant negative correlation with soil pH values (Table 6), indicating stronger binding of selenite ions at lower pH values, a phenomenon well established in the literature (Balistrieri and Chao 1987; Goldberg 2014a, b; Gabos et al. 2014).

**Table 5:** Parameters of the Langmuir and Freundlich models for Se(IV) in the six soils, Se(IV) in the six soils-Ca-Z, and Se(VI) in the six soils-Ca-Z

Soil	Langmuir constants								Freundlich constants								
	*q*_*m*_ (mg g^−1^)		*b*_*L*_ (L mg^−1^)		*R*^2^		*p*-value			*K*_*F*_ (mg g^−1^)(L/mg)^1/*n*^		*1*/*n*		*R*^2^		*p*-value	
Se(IV) in the six soils																	
1	0.26		0.094		0.918		< 0.001			5.32		0.382		0.842		< 0.01	
2	0.25		0.068		0.933		< 0.001			4.00		0.545		0.996		< 0.001	
3	0.31		0.116		0.911		< 0.001			5.90		0.391		0.974		< 0.01	
4	0.15		0.080		0.899		< 0.01			3.37		0.556		0.915		< 0.001	
5	0.11		0.095		0.967		< 0.001			3.18		0.507		0.924		< 0.001	
6	0.31		0.048		0.905		< 0.001			3.65		0.645		0.989		< 0.001	
Se(IV) in the six soils-Ca-Z																	
1		0.31		0.206		0.963		< 0.001			6.38		0.384		0.927		< 0.001
2		0.27		0.060		0.904		< 0.001			3.95		0.568		0.996		< 0.001
3		0.33		0.106		0.926		< 0.001			5.74		0.419		0.993		< 0.001
4		0.20		0.056		0.900		< 0.01			3.48		0.561		0.988		< 0.001
5		0.20		0.090		0.912		< 0.001			4.27		0.459		0.987		< 0.001
6		0.24		0.054		0.938		< 0.001			3.34		0.648		0.980		< 0.001
Se(VI) in the six soils-Ca-Z																	
1		0.10		0.123		0.933		< 0.001			4.14		0.286		0.974		< 0.001
2		0.12		0.091		0.905		< 0.001			3.88		0.367		0.983		< 0.001
3		0.11		0.127		0.976		< 0.001			3.66		0.423		0.980		< 0.001
4		0.08		0.137		0.934		< 0.001			3.76		0.296		0.944		< 0.001
5		0.10		0.081		0.900		< 0.01			3.88		0.291		0.840		< 0.01
6		0.13		0.089		0.940		< 0.001			4.10		0.338		0861		< 0.01

**Table 6:** Significant correlation coefficients between the isotherm models parameters and the soil properties, at *p* < 0.05 (*N* = 6)

	pH	Fe_o_ (%)	Fe_d_ (%)	Mn_o_ (%)	Mn_d_ (%)	Al_o_ (%)	Al_d_ (%)
*q*_*m*_* Se(IV)-Z*	− 0.823	0.890	0.857	0.903	0.911		0.835
*B*_*L*_* Se(IV)-Z*							0.895
*R*_*L*_* Se(IV)-Z*			− 0.840				− 0.892
*1/n Se(IV)*			− 0.950				− 0.862
*K*_*F*_* Se(IV)*		0.927	0.957	0.977		0.908	
*1/n Se(VI)-Z*		− 0.845					
*K*_*F*_* Se(VI)-Z*			0.956		0.849		0.965

*Z* modified zeolite

The presence of Ca–zeolite increased *q*_*m*_ in almost all cases, indicating a slightly higher capacity of the treated soils to adsorb Se(IV), in accordance with findings of XRF spectra. The opposite trend that appeared for the *b*_*L*_ values possibly suggests a lower bonding strength in the presence of Ca–zeolite and points to that Se(IV) availability may be increased in soils amended with materials as the Ca modified zeolite. This is in accordance with XAFS analysis that verified a physisorption mechanism (outer-sphere complexes) for Se(IV) sorption onto Ca–zeolite.

The negative Δ*G*^o^ values, calculated from the bonding constant, indicate that under the experimental conditions, the sorption process of both Se species was spontaneous and all soils in the presence or not of Ca–zeolite provided sites that promoted Se adsorption (Table 4).

The parameters from the two isotherms, namely, *q*_*m*_ from the Langmuir isotherm and *KF* from the Freundlich isotherm, showed in many cases significant correlations with soil constituents (Table 6). The crystalline and amorphous of Fe, Al, and Mn oxides showed positive correlation with the parameters *q*_*m*_ and *KF* of Langmuir and Freundlich isotherms, that in the presence of Ca–zeolite became significant, supporting the role of free oxides (especially that of iron) on the geochemical behavior of selenite.

Selenate forms weak outer-sphere complexes with soil colloids and is thus poorly adsorbed by soils, resulting in environmental risks, with frequent contamination of aquifers when added as inorganic fertilizer. Thus, in the present study, adsorption was carried out only in the presence of modified zeolite and the experimental data described adequately by the two models. The *r*^2^ mean values were 0.93 for both Freundlich and Langmuir isotherms (Table 5). In contrast to the case of Se(IV), Se(VI) shows no significant correlations between the parameters of the equilibrium equations and soil characteristics, indicating that the presence of the modified mineral in the soil mixture played a major role in the adsorption of this species. At the same time, it confirms the fact that the adsorption of this species on soil colloids is extremely low and not controlled by metal oxides. The XAFS analysis showed that physisorption was the main mechanism for Se(VI) adsorption on the modified mineral without any change of species as it was also observed for Se(IV).

**Fig. 4:**
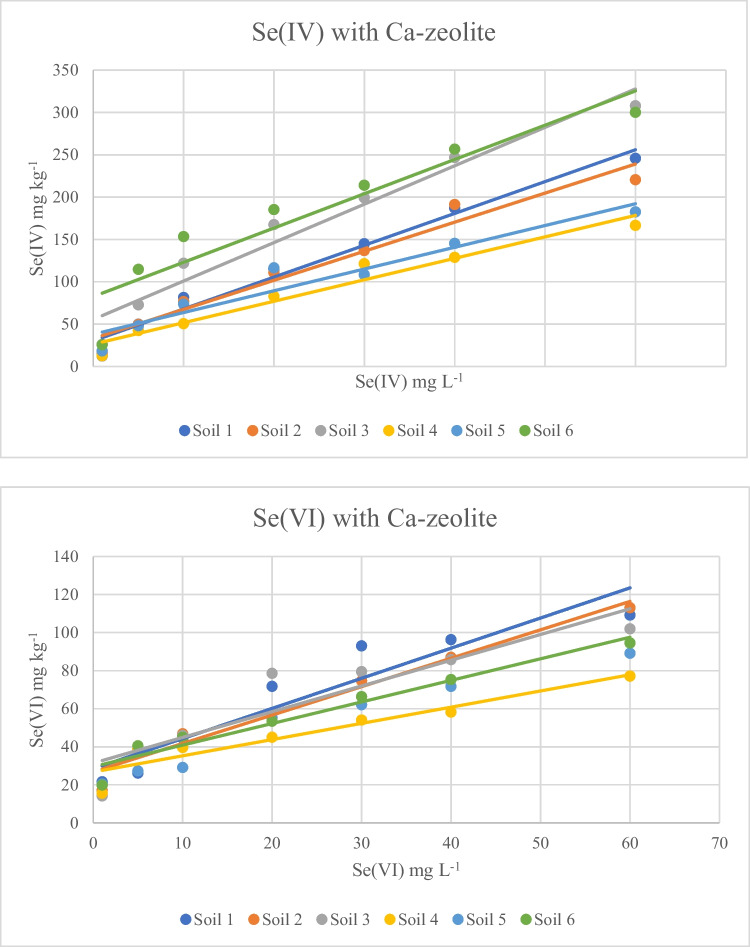
Se(IV) and Se(VI) adsorption on six soils-Ca-Z mixtures, at various initial Se(IV) concentrations. Contact time 24 h, agitation rate 125 rpm, sorbent/solution ratio 1 g/0.03 L, Se concentrations at start time from 1 to 60 mg/L, temperature 22 °C. Equilibrium solution pH values 6.0–6.5

**Fig. 5:**
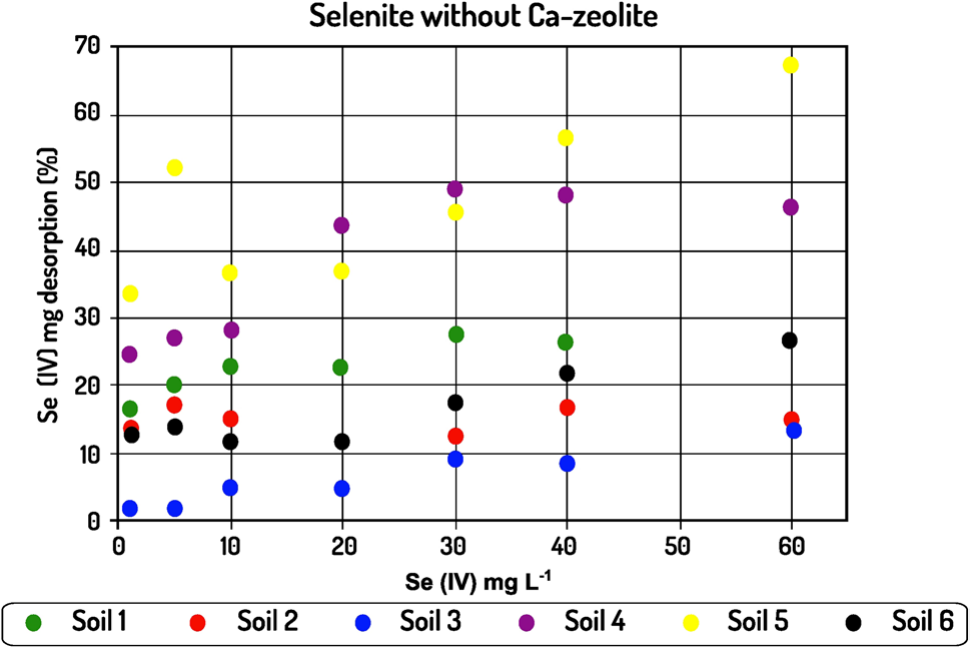
Se(IV) percentage desorption from the six soils, at various initial Se(IV) concentrations. Agitation rate 125 rpm, sorbent/solution ratio 1 g/0.03 L, temperature 22 °C

**Fig. 6:**
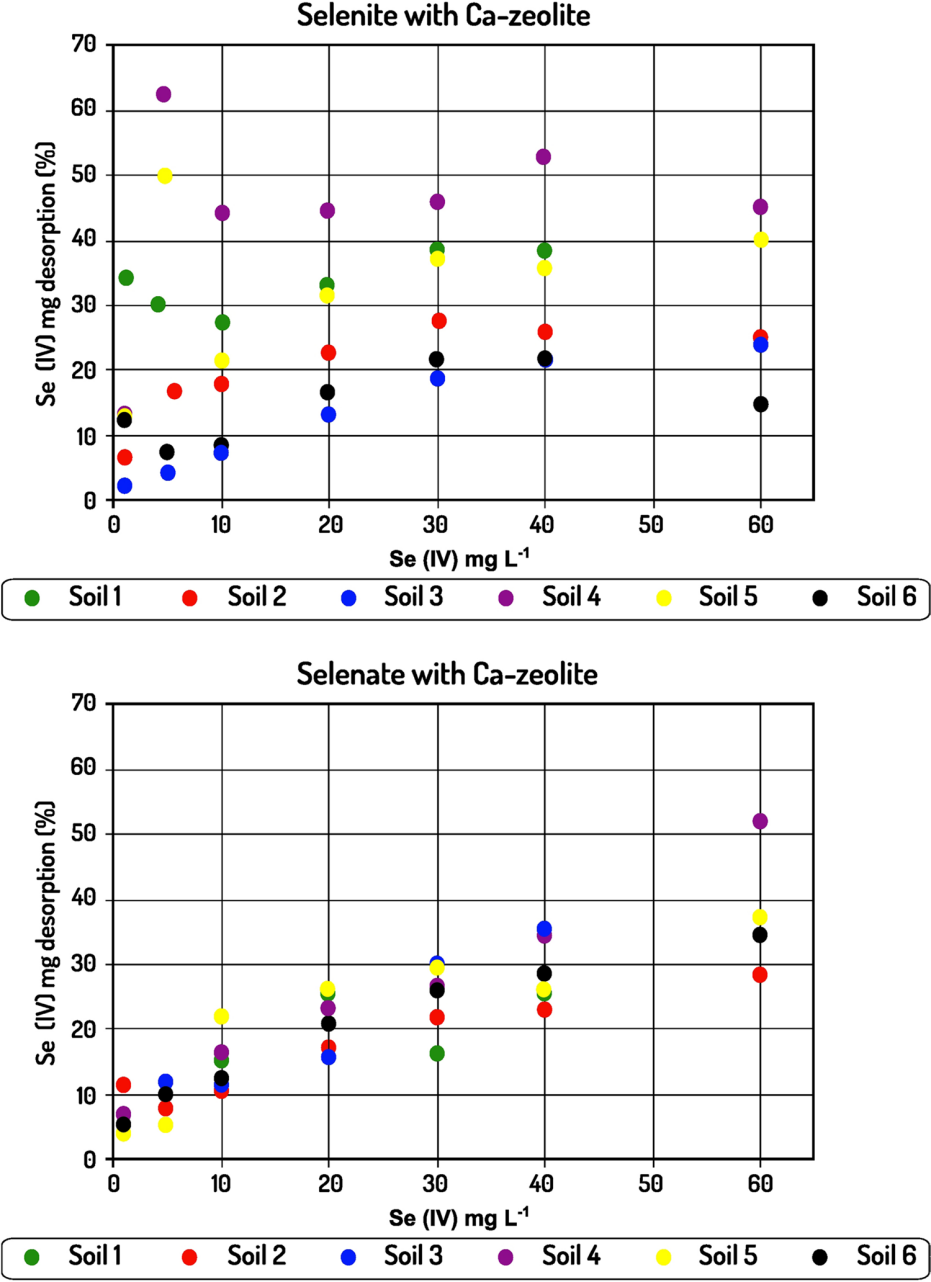
Se(IV) and Se(VI) percentage desorption percentage from the six soils-Ca-Z mixtures, at various initial Se(VI) concentrations. Agitation rate 125 rpm, sorbent/solution ratio 1 g/0.03 L, temperature 22 °C

The most important findings of the present study are that in the presence of Ca–zeolite not only did adsorption of Se, regardless of its species, described by the two models, for both acid and alkaline soils, but also that the formation of outer-sphere complexes on Ca-Z imply that Se can be readily available for plant uptake. Indeed, while in all cases the amount adsorbed was highest for selenite (Figs. 3, 4, 5, and 6), desorption percentages of selenate were lower or similar to those of selenite. This suggests that the presence of Ca–zeolite in the conditions of the present study made the desorption patterns of the two chemical species to be similar in both acid and alkaline soils, thus suggesting a possible solution to reduce the environmental risk due to the use of selenate inorganic fertilizers.

As presented in Table 4, the equilibrium parameter (*R*_*L*_) values for all soils and all treatments were always < 1. Moreover, *R*_*L*_ decreased as the initial selenium solution concentration increased. Constantino et al. (2017, 2018) support that the sorption process is feasible because *R*_*L*_ < 1, and that a decreasing trend of *R*_*L*_ values implies higher feasibility of sorption process as the initial concentration adsorbate increases. Vargas et al. (2011) report that decreasing *R*_*L*_ values as the initial adsorbate concentration in the solution increases indicate that adsorption is more favorable at high concentrations. Thus, the fact that *R*_*L*_ values for all initial selenium solution concentrations were < 1 and decreased as the concentration increased strongly indicates a feasible and favorable sorption process of Se(IV) on soils and of both Se species on soils treated with Ca–zeolite, and that their affinity for the adsorbing surface is higher at higher initial Se concentrations.

## Conclusions and future work

Following the “farm to fork” EU policy and acknowledging the necessity for improved nutritional value of the produced food/feed and the concurrent protection of the environment, the conducted study provided promising results in terms of both considerations. According to the results and discussion of the performed batch experiments, the incorporation of Ca-modified zeolite in different surface soils, typical of the Mediterranean agroecosystems, can improve selenite availability and can create adsorption sites for the retention of selenate in soils also regulating the desorption rate and thus potentially reducing the leaching of the oxyanion which otherwise would most likely end in subsurface aquifers. Especially for the low Se concentrations that are used for biofortification purposes, the results positively suggest that selenate can be applied to cultivated soils, whereas leaching can be greatly restricted. On the other hand, in case of Se-rich soils, the incorporation of Ca-modified zeolite may reduce the toxic hazards for the local ecosystems, protecting plants and consequently animals and human health.

The information provided in this study may be helpful for a better understanding of selenium behavior in soil environments, and by proposing a feasible method of CaCl_2_-modified natural zeolite incorporation in soils, selenium leaching may be reduced especially when selenate is the predominant species. Following this, future works should be focused on the development of a commercial Se fertilization product enriched in Ca–zeolite, and since soil–plant interactions and rhizosphere environment control the uptake of nutrients by plants, field experiments should be performed to conclude on the results of the present study and to test the efficiency of the proposed Ca–zeolite modification for agronomic use.

## Data Availability

The data that support this study will be shared on request to the corresponding author.
